# The effects of exogenous surfactant administration on ventilation-induced inflammation in mouse models of lung injury

**DOI:** 10.1186/1471-2466-13-67

**Published:** 2013-11-20

**Authors:** Valeria Puntorieri, Josh Qua Hiansen, Lynda A McCaig, Li-Juan Yao, Ruud AW Veldhuizen, James F Lewis

**Affiliations:** 1 Department of Physiology & Pharmacology, Western University, London, Ontario, Canada; 2 Department of Medicine, Western University, London, ON, Canada; 3 Lawson Health Research Institute, London, ON, Canada

**Keywords:** Acute lung injury, Mechanical ventilation, Exogenous surfactant, Systemic inflammation

## Abstract

**Background:**

Mechanical ventilation (MV) is an essential supportive therapy for acute lung injury (ALI); however it can also contribute to systemic inflammation. Since pulmonary surfactant has anti-inflammatory properties, the aim of the study was to investigate the effect of exogenous surfactant administration on ventilation-induced systemic inflammation.

**Methods:**

Mice were randomized to receive an intra-tracheal instillation of a natural exogenous surfactant preparation (bLES, 50 mg/kg) or no treatment as a control. MV was then performed using the isolated and perfused mouse lung (IPML) set up. This model allowed for lung perfusion during MV. In experiment 1, mice were exposed to mechanical ventilation only (tidal volume =20 mL/kg, 2 hours). In experiment 2, hydrochloric acid or air was instilled intra-tracheally four hours before applying exogenous surfactant and ventilation (tidal volume =5 mL/kg, 2 hours).

**Results:**

For both experiments, exogenous surfactant administration led to increased total and functional surfactant in the treated groups compared to the controls. Exogenous surfactant administration in mice exposed to MV only did not affect peak inspiratory pressure (PIP), lung IL-6 levels and the development of perfusate inflammation compared to non-treated controls. Acid injured mice exposed to conventional MV showed elevated PIP, lung IL-6 and protein levels and greater perfusate inflammation compared to air instilled controls. Instillation of exogenous surfactant did not influence the development of lung injury. Moreover, exogenous surfactant was not effective in reducing the concentration of inflammatory cytokines in the perfusate.

**Conclusions:**

The data indicates that exogenous surfactant did not mitigate ventilation-induced systemic inflammation in our models. Future studies will focus on altering surfactant composition to improve its immuno-modulating activity.

## Background

Pulmonary surfactant is a mixture of phospholipids, surfactant-associated proteins and neutral lipids which has an important role in the lung in both host defence mechanisms such as modulating pulmonary inflammation and in stabilizing the alveoli by reducing surface tension [1,2]. Both biophysical and immuno-modulatory properties of endogenous surfactant are essential for normal lung function. Importantly, both properties are severely impaired during the course of acute lung injury (ALI) [3,4].

ALI is a life threatening condition characterized by bilateral pulmonary infiltrates on chest radiograph, alveolar edema and hypoxemia [5]. Mortality is approximately 30-40%, with the main cause of death resulting from multiple organ failure (MOF) rather than respiratory failure. The former is thought to develop in large part due to the release of inflammatory mediators from the lung into the circulation thereby contributing to excessive systemic inflammation. This, in turn, causes MOF and death [6-8].

The main supportive therapy required to maintain adequate oxygenation for patients with ALI is mechanical ventilation (MV). Unfortunately, this intervention is also an important component of the complex pathophysiology of ALI, since it can increase pulmonary inflammation and contribute to the development of the associated systemic inflammation leading to MOF [9-13]. A pharmacological therapy capable of mitigating the specific inflammatory effects of MV thereby reducing the contribution of the lung to the systemic inflammation is needed. Based on the known properties of surfactant within the lung, the current study investigated on such potential therapy namely exogenous surfactant administration.

Exogenous surfactant has been investigated as a possible therapy for ALI in many experimental and clinical studies [14-17]. Traditionally surfactant treatment has been administered to improve the biophysical function of this material within the lung. Although extensive research has shown improvements in physiological and biophysical outcomes following surfactant treatment, there was no effect on mortality [18]. Contrasting this extensively investigated approach, only a limited number of studies have evaluated surfactant with the aim to down-regulate the systemic inflammation associated with ALI and MV. Previous studies in our laboratory demonstrated that elevated endogenous surfactant pool sizes *prior to MV* attenuated the development of pulmonary and systemic inflammation in animal models where injurious MV was applied to normal lungs [19] or conventional ventilation was applied to lungs with a pre-existing injury (lipopolysaccharide-induced ALI) [20]. Whether exogenous surfactant can mirror these observations obtained with elevated endogenous surfactant is not known. It was therefore hypothesized that administration of exogenous surfactant *prior* to MV would reduce the *systemic inflammation* associated with lung injury.

To test this hypothesis, two separate mouse models were utilized: i) a model of mechanical ventilation in animals with otherwise normal lungs and ii) a model of acid-induced lung injury followed by MV. For both experiments, exogenous surfactant was administered *prior to* MV, and the ventilation was performed *ex vivo* using an isolated and perfused mouse lung (IPML) setup. The inflammatory mediators released by the lungs into the circulation were collected (via left ventricle) in perfusate and re-circulated (via pulmonary artery) throughout MV. This *ex vivo* circulatory system in the IPML setup allowed us to isolate the contribution of mechanically ventilated lungs to the systemic system, with perfusate representing a surrogate of systemic inflammation.

## Methods

### Experimental design and ethics statement

A total of 36 male 129X1/SVJ mice (Jackson Laboratories, Bar Harbor, Me., USA) were utilized for two separate animal experiments. All procedures were approved by the Animal Use Subcommittee at Western University (Permit Number: 2010–272) and, whenever necessary, adequate anesthetic regimen was used to minimize suffering. For both experiments, mice were allowed to acclimatize for a minimum period of 72 hours in an animal facility, during which time they were allowed free access to water and standard chow.

In order to test our hypothesis of an anti-inflammatory role of surfactant toward the effects of MV, administration of exogenous surfactant was performed in two separate models of lung injury: *experiment 1* involved the use of MV only and *experiment 2* involved the use of intra-tracheal (i.t.) instillation of hydrochloric acid (HCl) followed by conventional MV.

In *experiment 1*, mice were anaesthetized and subsequently randomized to either exogenous surfactant administration or no treatment. After the completion of the i.t. surfactant instillation, mice were connected to the IPML setup and exposed immediately following re-perfusion to MV with a tidal volume (Vt) of 20 ml/kg, a positive end expiratory pressure (PEEP) of 3 cmH_2_O, and a respiratory rate (RR) of 30 breaths/min. This resulted in the randomization of a total of 12 mice to one of the two experimental conditions: i) No Treatment group or ii) bLES group.

In *experiment 2,* a total of 24 male 129X1/SVJ mice were anaesthetized and then randomized to receive an intra-tracheal instillation of HCl or air. Four hours after the development of acid-induced lung injury, mice were randomized to receive an intra-tracheal exogenous surfactant administration (or no treatment) before *ex vivo, in situ* MV. The IPML setup was used to ventilate these animals with the following ventilation parameters: Vt = 5 ml/kg, PEEP = 3 cmH_2_O, RR = 60 breaths/min. This resulted in the following experimental conditions: i) air + no treatment; ii) air + bLES; iii) acid + no treatment; iv) acid + bLES.

### Intra-tracheal hydrochloric acid instillation

Mice were randomized to receive either an intra-tracheal (i.t.) administration of HCl or air as a control, as previously described [9]. Briefly, mice were anesthetised with an intra-peritoneal injection of ketamine (130 mg/kg; Sandoz, Quebec, Que., Canada) and xylazine (6 mg/kg; Bayer, Toronto, Ont., Canada). Once the proper depth of anesthesia was reached, mice were positioned dorsally on a vertical stand and their trachea was intubated with a 20-gauge catheter coupled with a fiber-optic stylet (BioLite intubation system for small rodents, BioTex, Inc., Houston, Tex., USA). Animals randomized to the acid instillation group were given 50 μl of 0.05 Ν HCl in a drop-wise fashion through the endotracheal tube. Animals randomized to the control group were intubated as described and allowed to breathe spontaneously through the tube. The total procedure took approximately 5 minutes. Mice were then extubated, positioned on a horizontal inclined stand and administered sub-cutaneous injections of buprenorphine (0.05-0.1 mg/kg) and 1 ml of sterile normal saline. Subsequently, mice were returned to the cage and allowed to recover for 4 hours with free access to water and food. Mice were carefully monitored during the 4 hours recovery period.

### Intra-tracheal surfactant instillation

Mice were anesthetised with an intra-peritoneal (i.p.) injection of ketamine (130 mg/kg) and xylazine (6 mg/kg). Animals were then positioned dorsally on a vertical rodent stand and the trachea was intubated trans-orally with a 20-gauge catheter coupled with a fiber-optic stylet (BioLite intubation system for small rodents, BioTex, Inc., Houston, Tex., USA). Mice randomized to the surfactant administration group were given 50 mg/kg bLES (BLES Biochemicals, London, Ont., Canada) in a drop wise fashion through the endotracheal tube. This natural, bovine lipid extracted surfactant is composed of approximately 97% phospholipids, 3% neutral lipids, and about 1% by weight proteins [21]. After the surfactant was spontaneously inhaled by the animals, mice were extubated and positioned on a horizontal inclined stand. To allow for peripheral surfactant distribution, based on preliminary experiments, mice were allowed to spontaneously breathe for 12–15 minutes before MV. Animals randomized to the no treatment group were intubated as described and allowed to breathe spontaneously.

### Isolated and perfused mouse lung setup

Mice were ventilated for a total of 2 hours using the IPML setup. Following exogenous surfactant administration (or no treatment), the anesthetised mice were sacrificed with an additional i.p. injection of ketamine (200 mg/kg) and xylazine (10 mg/kg). A tracheostomy tube was then inserted and secured in the trachea, and the animals were subsequently connected to the IPML apparatus as described by Von Bethmann et al. [22]. Briefly, the heart and lungs were surgically exposed and the lungs were ventilated with a volume cycled, positive pressure ventilator (Flexivent, Scireq, Montreal, Que., Canada) with different ventilation strategies as described in detail under the *experimental design* section. Perfusate (RPMI lacking phenol red + 2% w/v low endotoxin grade Bovine Serum Albumin; Sigma, St. Louis, Mo., USA) was circulated into the pulmonary vasculature through a catheter inserted in the pulmonary artery and collected by a second catheter in the left ventricle. Once the lungs were cleared of all the blood, perfusate was delivered in a re-circulating fashion (rate 1 ml/min) during the 2 hours of MV. One milliliter of perfusate was collected at baseline (time 0, immediately after vascular clearing and before perfusate re-circulation) and every 30 minutes of MV thereafter. Samples were frozen and stored at −80°C for subsequent measurement of inflammatory mediators. Physiological parameters such as peak inspiratory pressure (PIP) and perfusion pressure were monitored throughout ventilation utilizing Chart v.4.12 software (AD Instruments, Castle Hill, Australia).

### Surfactant and total lung lavage protein measurements

Immediately after MV using the IPML setup, lungs were lavaged with 3 × 1 ml aliquots of 0.9% NaCl solution with each aliquot instilled and withdrawn 3 times. The total lavage volume was recorded and average recoveries of lavage fluid were 2.7 mL and 2.8 mL for *experiment 1* and *experiment 2*, respectively. Total lavage was then immediately centrifuged at 380 g for 10 min. at 4°C to remove the cellular component, and the collected supernatant was termed total surfactant (TS). A 1 ml aliquot of TS was stored at −80°C for cytokine and protein analysis. In order to separate the small aggregate sub-fraction (SA) from the large aggregate (LA) sub-fraction, 1 ml of TS was centrifuged at 40,000 g for 15 min at 4°C. The LA pellet was then re-suspended in 0.3 ml of 0.9% NaCl, while the supernatant represented the SA fraction. The leftover volume of TS was used for analysis of total surfactant pool size. TS, LA and SA were frozen and stored at −80°C.

Measurement of the phospholipid content in TS, LA and SA was performed by phosphorous assay on chloroform-methanol extracted samples, as previously described [23,24]. Total protein content in lavage was assessed using a Micro BCA protein assay kit (Pierce, Rockford, Ill., USA) according to manufacturer’s instructions.

### Biophysical functional analysis of surfactant

LA sub-fractions from animals within each experimental group were pooled together for functional analysis. An aliquot from each pooled sample was utilized to measure the total phospholipid content by phosphorous assay, while the remaining pooled LA was centrifuged at 40,000 g for 15 min at 4°C. The supernatant was then discarded and the purified LA pellet re-suspended in a buffer solution (1.5 mM CaCl_2_, 5 mM TRIS) to a final phospholipid concentration of 5 mg/ml. The surface activity of the LA samples was assessed using a computer-controlled captive bubble surfactometer (CBS, 3 runs for each pooled sample) as previously described [25,26].

### Measurement of inflammatory mediators

Interleukin-6 (IL-6) levels were measured in aliquots of lung lavage and in perfusate aliquots obtained at different time points using an enzyme-linked immunosorbent assay (ELISA) kit following manufacturer’s instructions (BD Biosciences, San Diego, CA., USA). A broader array of inflammatory mediators was measured in perfusate samples collected at the end of MV using a Milliplex Map mouse cytokine/chemokine panel (MPXMCYTO-70 K-12; Millipore Corporation, Billerica, MA, USA) for the following 12 analytes: eotaxin, granulocyte colony-stimulating factor (G-CSF), granulocyte-macrophage colony-stimulating factor (GM-CSF), IL-1β, IL-6, IL-13, interferon-γ-induced protein 10 (IP-10), keratinocyte chemoattractant (KC), lipopolysaccharide-induced CXC chemochine (LIX), monocyte chemotactic protein-1 (MCP-1), macrophage inflammatory protein 2 (MIP-2) and tumor necrosis factor-alpha (TNF-α). Samples were analyzed utilizing the Luminex® xMAP® detection system on the Luminex^100^ (Linco Research, St. Charles, Mo., USA) according to the manufacturer’s instructions. Perfusate samples collected at the end of MV in *experiment 2* were further analyzed for eicosanoids levels (8-isoprostane, prostaglandin E_2_, leukotriene B_4_, thromboxane B_2_) using colorimetric competitive enzyme immunoassay (EIA) kits (Cayman Chemical Company, Ann Arbor, MI, USA) according to manufacturer’s instructions.

### Statistical analysis

All data are expressed as mean ± standard error of the mean (SEM). Statistical analyses were performed using the GraphPad Prism statistical software (GraphPad Software, Inc., La Jolla, CA., USA). Data were analysed with a t-test or one way ANOVA with a Tukey’s post hoc test when appropriate (*experiment 1)*. For *experiment 2,* a two-way ANOVA (variables: presence of primary insult and treatment effects) followed by a one-way ANOVA with a Tukey’s post hoc test was used to analyse the data. A repeated measures two-way ANOVA was performed when appropriate with a Bonferroni post hoc test. P < 0.05 was considered statistically significant.

## Results

### Experiment 1

In experiment 1 the effects of exogenous surfactant administration on lung and systemic inflammation during MV of otherwise normal lungs were determined. Peak inspiratory pressure (PIP) was recorded throughout MV. PIP ranged between 20.62 ± 1.6 cmH_2_O and 22.6 ± 2.7 cmH_2_O for the No Treatment group (time 0 and time 120 min, respectively) and varied between 22.6 ± 2.7 cmH_2_O and 26.3 ± 2.7 cmH_2_O for the bLES group (time 0 and time 120 min, respectively). Exogenous surfactant administration did not reduce PIP values in the surfactant treated group compared to No Treatment. Perfusion pressure was also monitored throughout MV and maintained between 4 and 6 mmHg for both groups (data not shown).

#### Lavage analysis

Results reflecting local inflammation, as assessed by pulmonary permeability changes and inflammatory markers are shown in Table 1. The total protein content and IL-6 levels in lung lavage collected at the end of MV were not affected by surfactant treatment, with no statistically significant differences noted in these values between bLES treated and non-treated groups. Recoveries of lung lavage fluid were not statistically significant between groups (data not shown).

**Table 1:** ***Experiment 1: *** **Total protein levels and IL-6 concentrations in lung lavage at the end of MV**

	**Mechanical ventilation**	
	**No treatment**	**bLES**
**Total lavage protein (mg/kg body weight)**	13.4 ± 1.2	34.6 ± 18.9
**Lavage IL-6 (pg/mL)**	136.8 ± 31.4	474.1 ± 233.8

Values are expressed as mean ± SEM; n = 6 per group.

**Figure 1:**
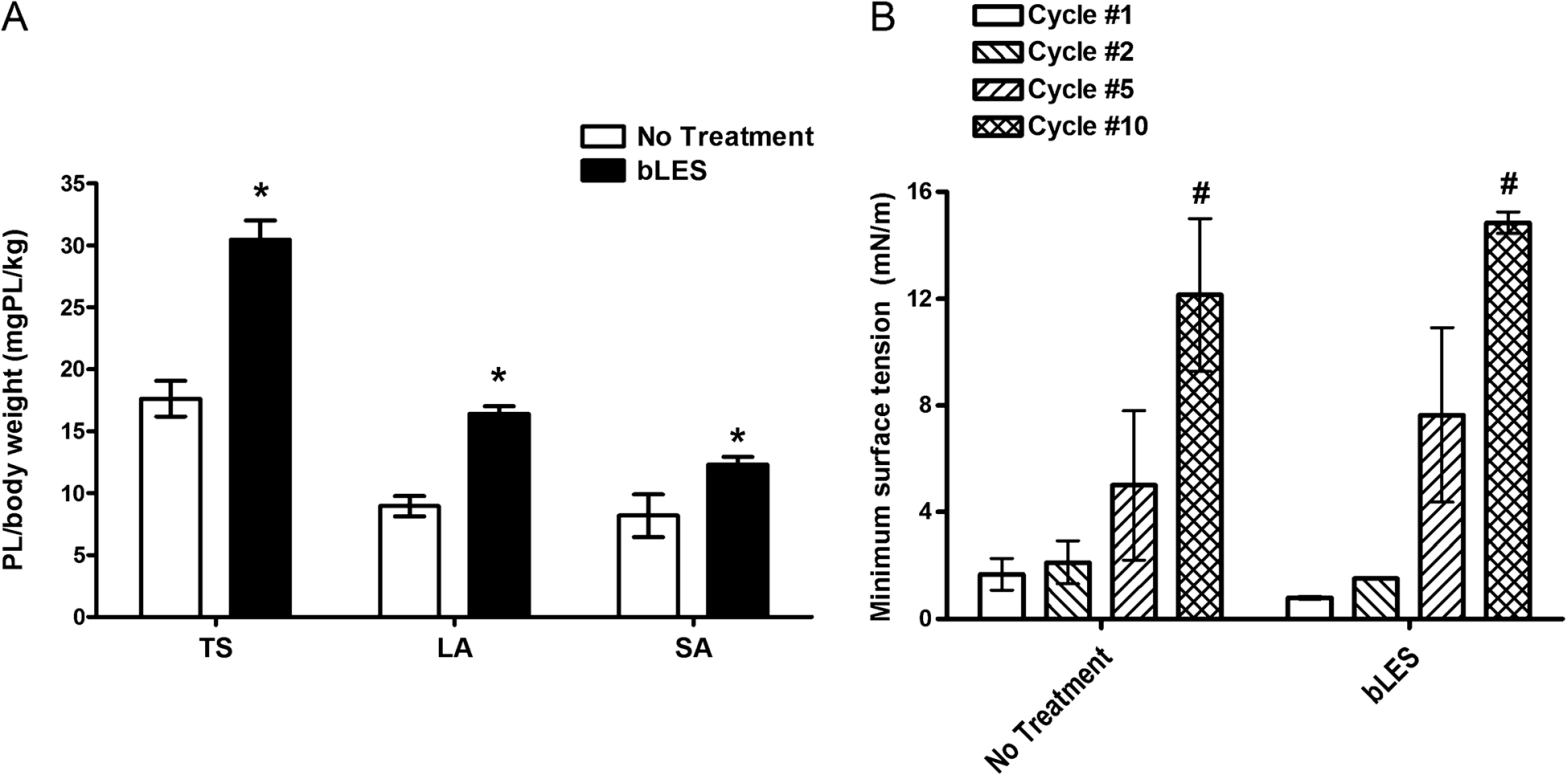
***Experiment 1: *Surfactant recovery in lung lavage and surface activity of LA. A**: surfactant pool size of TS, LA and SA sub-fractions measured by phosphorous assay. Data are expressed as amount of phospholipids/kg body weight. Within each sub-fraction, *p < 0.05 vs the No Treatment condition. **B**: minimum surface tension of pooled LA samples during different dynamic compression-expansion cycles. #p < 0.05 versus cycle 1 and 2 within each experimental condition. Values are expressed as mean ± SEM.; n = 6 per group.

Surfactant pool sizes of TS, LA and SA sub-fractions isolated from lung lavage for the two groups are shown in Figure 1A. As expected, TS pools were significantly higher in the bLES treated group compared to No Treatment mice. Similarly to TS values, LA and SA pools were significantly higher in the bLES group compared to the No Treatment group (Figure 1A).

The functional activity of the LA samples measured during four different dynamic compression-expansion cycles is shown in Figure 1B for each experimental group. No significant differences in surface tension were found between bLES treated and No Treatment mice for any of the cycles. Within each group, the minimum achievable surface tension was significantly higher during cycle 10 compared with cycles 1 and 2 (Figure 1B).

#### Perfusate analysis

**Figure 2:**
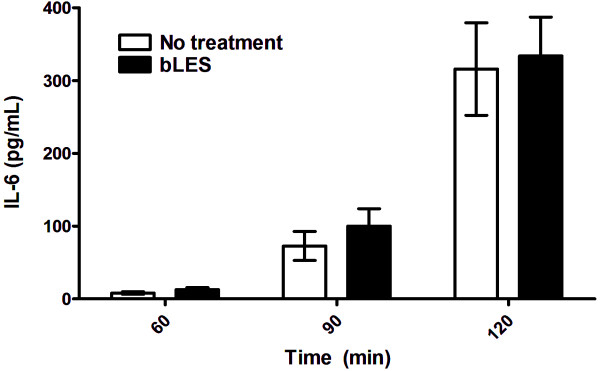
***Experiment 1: *IL-6 levels measured in lung perfusate at 60, 90 and 120 min.** Values are expressed as mean ± SEM.; n = 6 per group.

The concentration of IL-6 was measured in perfusate samples in order to assess the effects of exogenous surfactant on the development of systemic inflammation (Figure 2). IL-6 levels were not detectable within the first 30 minutes of MV (time 0 and 30 min; data not shown). A gradual increase in perfusate IL-6 was measured at 60 and 90 minutes in both groups; however, there was no statistically significant difference in this cytokine level between bLES treated and No Treatment mice at any time point throughout MV.

Perfusate concentrations of 11 cytokines/chemokines measured at the end of MV by multiplex assay are shown in Table 2. Perfusate IL-13 levels were not detectable (data not shown). There was no statistically significant effect of exogenous surfactant administration on cytokines/chemokines concentrations in perfusate, with no differences between No treatment and bLES groups.

**Table 2:** ***Experiment 1: *** **Cytokine and chemokine analysis in lung perfusate at the end of MV**

	**Mechanical ventilation**	
**Mediator (pg/ml)**	**No treatment**	**bLES**
Eotaxin	42.8 ± 3.6	42.7 ± 6.5
G-CSF	9.5 ± 2.0	11.7 ± 2.8
GM-CSF	1.6 ± 1.6	5.1 ± 2.4
IL-6	520.6 ± 117.2	463.0 ± 75.2
IL-1 β	0.8 ± 0.4	0.6 ± 0.3
KC	868.6 ± 254.3	853.8 ± 222.6
LIX	71.3 ± 14.8	66.1 ± 11.4
MCP-1	12.2 ± 2.7	7.5 ± 1.8
MIP-2	753.9 ± 193.8	658.5 ± 167.8
TNF-α	23.4 ± 7.8	17.4 ± 7.0
IP-10	39.9 ± 5.4	37.8 ± 6.2

G-CSF = granulocyte colony-stimulating factor, GM-CSF = granulocyte-macrophage CSF, IL-6 = interleukin-6, IP-10 = interferon-γ-induced protein 10, KC = keratinocyte chemoattractant, LIX = lipopolysaccharide-induced CXC chemokine, MCP-1 = monocyte chemotactic protein-1, MIP-2 = macrophage inflammatory protein 2 and TNF-α = tumor necrosis factor-alpha.

Data are expressed as mean ± SEM; n = 6 per group.

### Experiment 2

**Figure 3:**
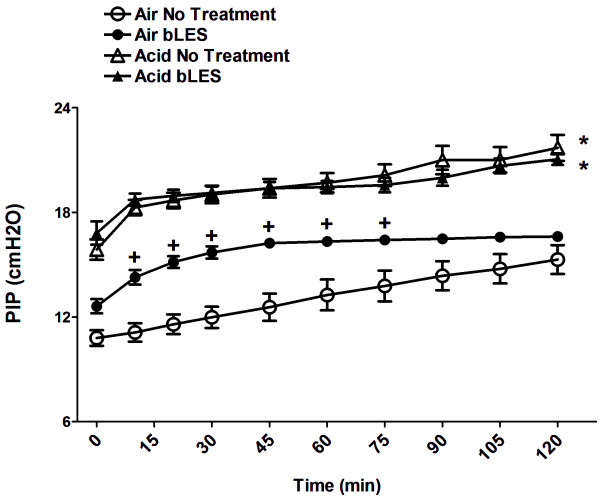
***Experiment 2: *Peak Inspiratory Pressure (PIP) was measured over the course of MV.** Values are expressed as mean ± SEM. +p > 0.05 versus Air No Treatment at the specific time point indicated, *p < 0.05 versus the respective Air control at each time point; n = 6 per group.

In experiment 2, the effect of exogenous surfactant on systemic inflammation during MV was assessed in the presence of a pre-existing acid-induced lung injury/inflammation. Physiological parameters such as peak inspiratory pressure and perfusion pressure were monitored throughout ventilation as in experiment 1, and PIP values are shown in Figure 3. Although all experimental groups were exposed to the same ventilation strategy, the peak inspiratory pressure was significantly higher in Acid injured mice compared to the respective Air groups (Acid No Treatment vs Air No Treatment; Acid bLES vs Air bLES). Exogenous surfactant administration led to a significant increase in PIP values during the first hour of MV (10 to 75 min) in the Air bLES group compared to Air No Treatment group and, importantly, did not reduce PIP values in the Acid bLES group compared to Acid No Treatment group at any time point. Perfusion pressure was monitored during MV and maintained between 5 and 7 mmHg for all groups (data not shown).

#### Lavage analysis

Lung permeability, as reflected by total protein content in lung lavage (Table 3), was significantly higher in the acid injured animals versus the air control groups, whether they were given surfactant or not (Acid No Treatment vs Air No Treatment; Acid bLES vs Air bLES). No significant difference was noted between Air bLES versus Air No Treatment and Acid bLES versus Acid No Treatment. Similar results were observed for IL-6 concentration in lung lavage (Table 3). Acid–instilled animals showed greater IL-6 levels in lavage compared to the respective air-instilled controls. Exogenous surfactant did not affect lavage IL-6 levels in both air groups (Air bLES vs Air No Treatment); however, there was a significantly higher cytokine concentration in the lavage of Acid bLES mice compared to the Acid No Treatment group. Recoveries of lung lavage fluid were not statistically significant between groups (data not shown).

**Table 3:** ***Experiment 2: *** **Total protein levels and IL-6 concentrations were measured in lung lavage at the end of MV**

	**Air**		**Acid**	
	**No treatment**	**bLES**	**No treatment**	**bLES**
**Total lavage protein (mg/kg body weight)**	46.3 ± 8.1	32.8 ± 5.6	215.4 ± 21.1*	194.3 ± 26.9*
**Lavage IL-6 (pg/mL)**	237.8 ± 72.9	635.1 ± 120.2	5034.9 ± 653.4*	6775.5 ± 1476.1*^,#^

Data are expressed as mean ± SEM; n = 6 per group. *p < 0.05 versus the respective Air control, #p < 0.05 versus Acid no Treatment.

**Figure 4:**
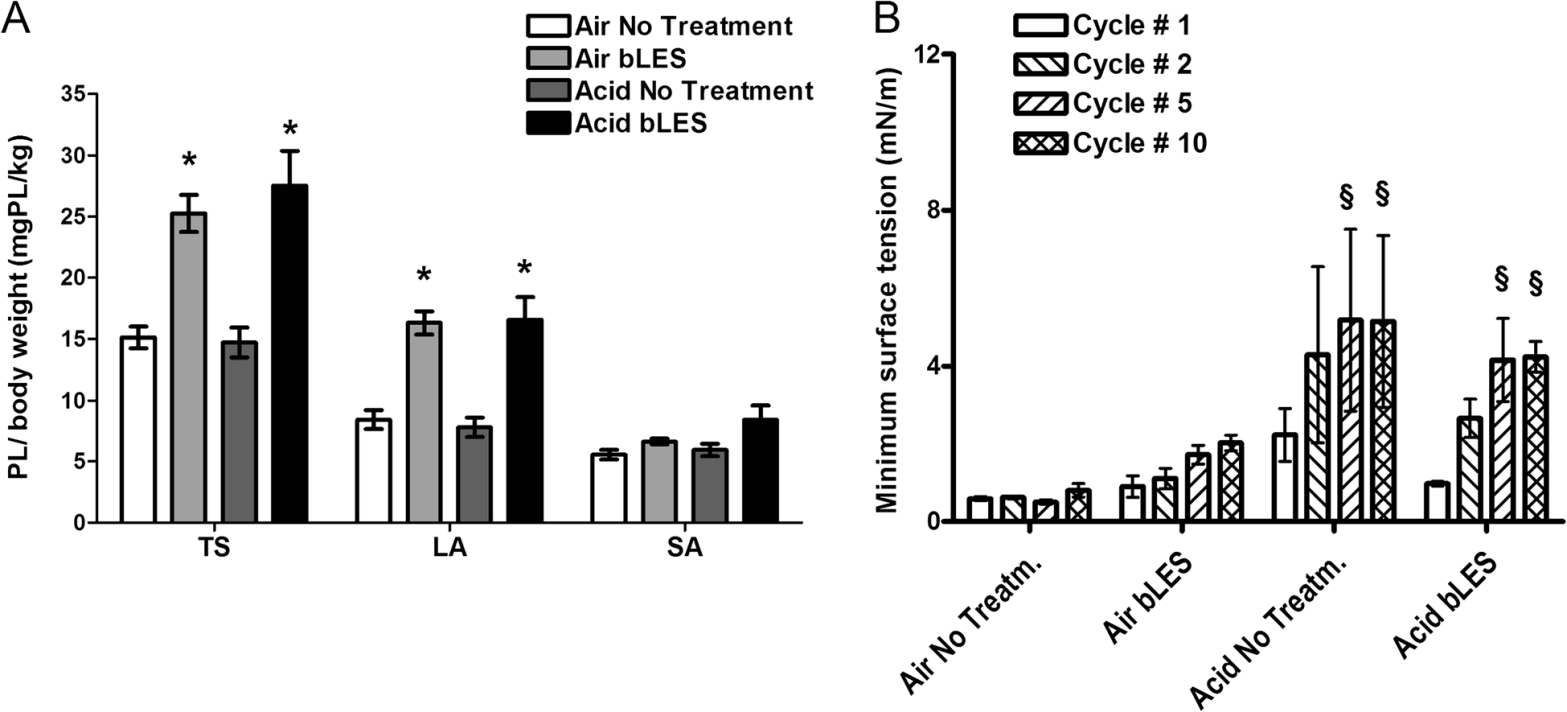
***Experiment 2: *Surfactant recovery in lung lavage and surface activity of crude LA. A**: surfactant pool size of TS, LA and SA sub-fractions measured by phosphorous assay. Data are expressed as amount of phospholipids/kg body weight. Within each sub-fraction,*p < 0.05 versus the respective No Treatment condition. **B**: surface tension of pooled LA samples during different dynamic compression-expansion cycles. §p < 0.05 versus cycle 1 within each experimental condition. Values are expressed as mean ± SEM; n = 6 per group.

Surfactant sub-fractions and the surface activity of isolated LA are shown in Figures 4A and B respectively. Acid instillation did not change TS, LA and SA pool sizes compared to their respective Air control groups (Figure 4A). This was similar for both not treated and surfactant treated groups. As expected and observed in experiment 1, total surfactant and LA values were significantly higher in surfactant treated groups than non-surfactant treated controls (Air bLES vs Air No Treatment; Acid bLES vs Acid No Treatment). There was no difference in SA values among the various experimental groups.

There were no statistically significant differences noted between any of the experimental groups in the biophysical activity of the LA samples (Figure 4B). Within some of the groups, however, significant differences in surface tension were measured between the different dynamic cycles. In particular, surface tension was significantly higher during compression-expansion of cycles 5 and 10 when compared to cycle 1 within the acid instilled groups (in both Acid No Treatment and Acid bLES). LA from the Air No Treatment and Air bLES groups maintained low surface tension values throughout the 10 dynamic compression-expansion cycles.

#### Perfusate analysis

**Figure 5:**
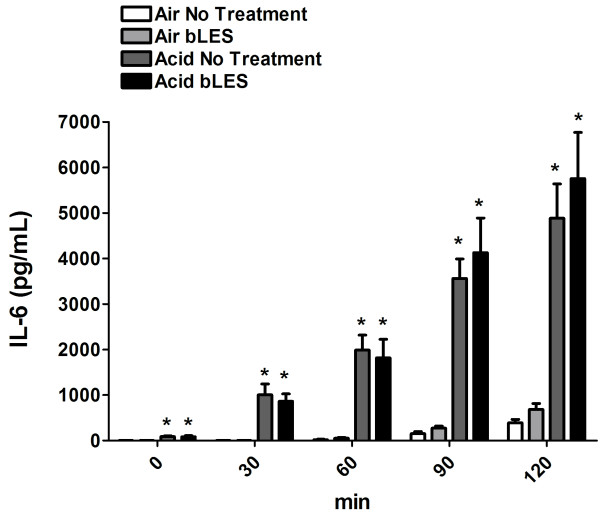
***Experiment 2: *IL-6 levels measured in lung perfusate at 0, 30, 60, 90, 120 min.** Data are expressed as mean ± SEM. *p < 0.05 versus respective Air control at each time point; n = 6 per group.

To test the hypothesis of a role for exogenous surfactant in down-modulating systemic inflammation in ALI, sequential lung perfusate samples, as a surrogate for systemic inflammation, were analyzed for IL-6 concentrations. As shown in Figure 5, there were significantly higher levels of IL-6 in the perfusate of acid-instilled mice compared to the respective air-instilled controls at every time point (0, 30, 60, 90, 120 min; Acid No Treatment vs Air No Treatment; Acid bLES vs Air bLES). Perfusate IL-6 levels were not significantly affected by exogenous surfactant administration, with no differences between Air bLES and Air No Treatment and no change between Acid bLES and Acid No treatment.

Lung perfusate samples collected at 120 min were further analyzed for a wider array of cytokines/chemokines. Among the 12 mediators measured (Table 4), IL-13 levels were not detectable (data not shown), while there were significantly greater levels of eotaxin, IL-6, KC, MIP-2 in acid-instilled animals compared to the respective air instilled control.

**Table 4:** ***Experiment 2: *** **Cytokine and chemokine levels measured in lung perfusate at the end of MV**

	**Air**		**Acid**	
**Mediator (pg/ml)**	**No treatment**	**bLES**	**No treatment**	**bLES**
Eotaxin	17.4 ± 1.3	25.7 ± 0.9	135.2 ± 13.6*	142.8 ± 15.9*
G-CSF	45.9 ± 10.5	59.4 ± 3.4	890.8 ± 75.8	1270.3 ± 187.8*
GM-CSF	0	0	13.4 ± 2.8	16.3 ± 2.2
IL-6	567.7 ± 62.7	1119.0 ± 99.1	8303.2 ± 323.6*	10720.1 ± 764.2*
IL-1 β	1.0 ± 0.40	0.2 ± 0.1	0.3 ± 0.1	3.2 ± 0.6
KC	579.2 ± 65.8	907.0 ± 75.4	3617.2 ± 174.3*	6212.7 ± 504.8*
LIX	113.7 ± 11.1	98.3 ± 13.0	284.6 ± 17.9	447.4 ± 31.9*
MCP-1	12.9 ± 1.8	30.9 ± 4.9	337.4 ± 26.2	558.0 ± 71.5*
MIP-2	584.4 ± 68.2	725.7 ± 41.9	1840.2 ± 76.7*	3113.4 ± 204.8*^,#^
TNF-α	63.4 ± 11.6	72.2 ± 7.0	119.7 ± 3.1	143.3 ± 8.1
IP-10	32.9 ± 3.6	40.8 ± 1.7	391.3 ± 80.8	283.3 ± 27.7

G-CSF = granulocyte colony-stimulating factor, GM-CSF = granulocyte-macrophage CSF, IL-6 = interleukin-6, IP-10 = interferon-γ-induced protein 10, KC = keratinocyte chemoattractant, LIX = lipopolysaccharide-induced CXC chemokine, MCP-1 = monocyte chemotactic protein-1, MIP-2 = macrophage inflammatory protein 2 and TNF-α = tumor necrosis factor-alpha.

Data are expressed as mean ± SEM; n = 6 per group. *p > 0.05 versus respective Air control, #p < 0.05 versus Acid No Treatment.

Overall, exogenous surfactant administration did not affect eotaxin, GM-CSF, IL-6, IL-1β, KC, TNF-α and IP-10 levels, with no statistical difference between the bLES and No Treatment group in both Air and Acid instilled mice.

A statistically significant increase of MIP-2 levels in the perfusate of Acid bLES mice was determined compared to Acid No Treatment, as well as significantly higher perfusate levels of G-CSF, LIX and MCP-1 in acid injured mice treated with surfactant compared to the Air bLES.

Finally, in order to further characterize the effect of exogenous surfactant administration on lung-derived mediators in perfusate, eicosanoids levels were also measured at the 120 min. time point (Table 5). Although increased levels of thromboxane B_2_ and prostaglandin E_2_ were recorded in the perfusate of acid-instilled animals compared to their respective Air controls, these changes did not reach statistical significance. Perfusate concentrations of 8-isoprostane were significantly higher in the acid injured groups compared to air controls. Surfactant treatment did not affect thromboxane B_2_ and 8-isoprostane concentrations. Prostaglandin E_2_ levels were significantly elevated only in the perfusate of Acid bLES mice compared to Air bLES controls. Leukotriene B_4_ levels were increased in the perfusate of Acid bLES mice but this difference failed to be statistically significant.

**Table 5:** ***Experiment 2: *** **Concentrations of prostaglandin E**_**2**_**, leukotriene B**_**4**_**, thromboxane B**_**2 **_**and 8-isoprostane measured in lung perfusate samples collected at the end of MV**

	**Air**		**Acid**	
**Mediator (pg/ml)**	**No treatment**	**bLES**	**No treatment**	**bLES**
Prostaglandin E_2_	14.3 ± 2.0	26.8 ± 3.9	147.4 ± 40.3	221.7 ± 89.2*
Leukotriene B_4_	14.4 ± 5.2	10.6 ± 5.3	13.1 ± 7.3	41.9 ± 12.3
Thromboxane B_2_	52.7 ± 10.1	67.8 ± 12.9	109.9 ± 24.8	134.9 ± 43.6
8-Isoprostane	11.3 ± 1.6	19.7 ± 3.0	47.2 ± 8.1*	70.4 ± 12.5*

Data are expressed as mean ± SEM; n = 6 per group. *p < 0.05 versus the respective Air control.

## Discussion

The overall objective of this study was to evaluate the anti-inflammatory effects of exogenous surfactant when administered prior to mechanical ventilation, either in the absence (experiment 1) or in the presence (experiment 2) of an initiating pulmonary insult. For both lung injury models, the IPML setup was utilized to specifically evaluate the contribution of ventilation to the development of systemic inflammation. MV of normal lungs resulted in the release of IL-6 (locally) into the airspace and several mediators (systemically) in the perfusate. Surfactant administration, however, was not effective in reducing the systemic inflammation associated with MV. Conventional ventilation of HCl instilled mice led to higher levels of both IL-6 and total protein in lavage, and significantly higher levels of pro-inflammatory mediators in perfusate without any effect of bLES instillation. Based on these results, it was concluded that administration of exogenous surfactant prior to MV does not reduce the systemic inflammation associated with lung injury in these models.

An important feature of the current study was to examine the effects of surfactant therapy in two different models. Analysis of the data showed important differences between the models, such as the degree of lung edema. Mechanical stretch of uninjured lungs did not affect lung permeability, whereas acid injured mice had increased total lavage proteins after two hours of MV. Another aspect that distinguishes the two models is represented by different levels of pulmonary and perfusate inflammation, which becomes particularly evident when comparing cytokine levels measured in the perfusate of the MV only, No Treatment group to the cytokine levels of the Acid No Treatment group. For example, MV of normal lungs caused a moderate increase in circulating Eotaxin, IL-6, KC and MIP-2, while acid instilled animals subjected to conventional MV had perfusate concentrations of these mediators that were at least two times greater. Given the greater inflammation characterizing the acid-injury model and the important role of lipid mediators in the development and progression of lung injury [27-32], eicosanoids levels were analyzed only on samples from experiment 2. Unambiguous conclusions about the effects of exogenous surfactant on systemic inflammation were therefore inferred from two experimental models with very different characteristics. This allowed us to rule out possible causes for the lack of efficacy of our treatment (such as presence/lack of pre-existing injury, specific effects of ventilation), and strengthened the understanding of the biological response.

Exogenous surfactant administration has been extensively investigated as a potential adjunctive therapy in acute lung injury [33-36]. The traditional approach with surfactant treatment has been to evaluate its efficacy in terms of physiological and biophysical improvements. Many experimental studies have in fact demonstrated that exogenous surfactant instilled after the onset of ventilation improved oxygenation, lung volume and compliance; moreover, it improved the surface tension reducing properties of the surfactant recovered from lung lavage subsequent to administration [15,37,38]. Nevertheless, despite this encouraging experimental evidence, clinical trials showed no improvement in mortality in surfactant treated patients even in the presence of an initial improvement in oxygenation [16,18]. It is possible that surfactant treatment in the previous studies was administered too late into ALI development; therefore earlier surfactant administration *prior to* or *at the onset* of MV could be more effective at mitigating disease progression. Since mortality can be improved by ameliorating ventilation – induced systemic inflammation [39], it was our interest to investigate whether exogenous surfactant could mitigate the effects of MV thereby down-modulating inflammation.

To our knowledge, the effect of surfactant on ventilation-induced release of inflammatory mediators in perfusate of an IPML model has been specifically addressed in two previous studies. Stamme and colleagues [40] showed elevated TNFα and IL-6 concentrations in the perfusate of surfactant treated animals compared to controls, in their mouse model of high pressure ventilation. In contrast, our group has shown a reduced level of inflammatory cytokines in perfusate due to elevated endogenous surfactant in an LPS model of injury [20]. Together with the current study in which surfactant did not impact inflammation in two models of injury, these data illustrate the complexity of surfactant treatment in which specific details of the experimental model may impact outcome. Furthermore, such details are obviously important to understand in the context of a potential clinical utilization of surfactant treatment to down-regulate systemic inflammation as well as to understand the mechanisms by which surfactant may affect inflammation.

Despite the lack of effect of surfactant treatment in our study, we speculate that mitigation of MV induced inflammation is still the best approach for an early intervention. Our data support earlier studies which showed that cytokines can be detected in perfusate rapidly after the onset of ventilation [22,41]. This loss of alveolar and systemic cytokine compartmentalization can lead to peripheral organ dysfunction, a condition of difficult clinical management. Therefore, targeting the lung with anti-inflammatory agents prior to MV may be a successful treatment option leading to improved outcomes. In this respect, surfactant could be utilized as a carrier for delivering lung specific anti-inflammatory agents prior to MV in future studies.

Along with the strengths of the present study, some limitations need to be addressed. Due to the lack of blood perfusion in the IPML setup, the lungs were not exposed during *ex vivo* MV to circulating soluble factors and immune cells which could have affected the progression of the injury. Moreover, *ex vivo* ventilation of perfused lungs did not favor the use of severe lung injury models, due to potential technical failure of the preparation. Consequently, the injury from ventilation was mild to moderate, thereby explaining the lack of change in surface tension or surfactant pool sizes. It is believed, however, that these limitations of the IPML setup were counter balanced by the advantage of specifically isolating lung-derived mediators released into the circulation, without the confounding contribution of systemic factors to the development of inflammation. Intra-tracheal instillation was also used for administering surfactant, ensuring the presence of large amounts of active material in the airspace before ventilation, as shown by higher levels of TS and LA in the lung lavage of treated animals. It should be acknowledged, however, that some inadequate distribution of surfactant might have occurred following instillation. Obstruction of smaller airways, with consequent heterogeneous lung inflation and regional over-distension might have been responsible for the increase in PIP (experiment 2, Air treated groups), and for the non-significant trend towards higher lavage IL-6 levels in the surfactant treated groups. Nevertheless, the instilled surfactant retained excellent biophysical properties as shown by the very low minimum surface tension achieved during dynamic compression-expansion of the crude LA. Overall, we believe that instillation did not account for the lack of efficacy of our treatment.

## Conclusions

In conclusion, this study expands the knowledge about exogenous surfactant treatment. It specifically focuses on the anti-inflammatory effects of a lung targeted therapy administered prior to MV on the development of systemic inflammation using two different mouse models. Although our data suggest a lack of efficacy for exogenous surfactant in down-modulating inflammation, future studies might focus on surfactant as a carrier for anti-inflammatory drugs or antibiotics in order to better interfere with ALI progression.

## Abbreviations

ALI: Acute lung injury; MV: Mechanical ventilation; MOF: Multi-organ failure; bLES: Bovine lipid extract surfactant; IPML: Isolated and perfused mouse lung; PIP: Peak inspiratory pressure; Vt: Tidal volume; PEEP: Positive end expiratory pressure; RR: Respiratory rate.

## Competing interests

The authors declare that they have no competing interests.

## Authors’ contributions

VP – Experimental procedures and design, data analysis, manuscript writing. JQH - Experimental procedures, data analysis, manuscript review. LAM - Experimental procedures. LJY - Experimental procedures. RAWV - Experimental design, data analysis, manuscript review. JFL - Experimental design, data analysis, manuscript review. All authors read and approved the final manuscript.

## Pre-publication history

The pre-publication history for this paper can be accessed here:

http://www.biomedcentral.com/1471-2466/13/67/prepub
